# Identification and characterization of larval and adult anopheline mosquito habitats in the Republic of Korea: potential use of remotely sensed data to estimate mosquito distributions

**DOI:** 10.1186/1476-072X-4-17

**Published:** 2005-07-13

**Authors:** Ratana Sithiprasasna, Won Ja Lee, Donald M Ugsang, Kenneth J Linthicum

**Affiliations:** 1 Department of Entomology, US Army Medical Component, Armed Forces Research Institute of Medical Sciences, 315/6 Rajvithi Road, Bangkok 10400, Thailand; 2 Laboratory of Medical Entomology, Department of Medical Zoology, National Institute of Health, 5, Nokbun-dong, Eunpyung-gu, Seoul, 122–701, Korea; 3 Space Technology Applications and Research Program, Asian Center for Research on Remote Sensing, Asian Institute of Technology, Pathumthani, Thailand; 4 Center for Medical, Agricultural & Veterinary Entomology, Agricultural Research Service, United States Department of Agriculture, P.O. Box 14565, 1600/1700 S.W. 23^rd ^Drive, Gainesville, Florida 32604, USA

## Abstract

**Background:**

*Plasmodium vivax *malaria reemerged in the Republic of Korea in 1993, with more than 2,000 cases reported in the northwestern part of the country over the last 10 years. To better assess the risk of malaria transmission we conducted a surveillance study to identify and characterize the habitats that produce potential *Anopheles *vector mosquitoes. Immature and adult mosquito collection data were incorporated into a Geographic Information System (GIS) along with remotely sensed satellite imagery, and imagery classified to land use to determine if remote sensing data could be used to estimate mosquito habitats.

**Results:**

More than 2,100 anopheline larvae were collected and mapped from 186 immature habitats, which were categorized into 9 types. *Anopheles sinensis *was the most commonly collected species, representing more than 97% of the specimens, followed by *Anopheles pullus *1.0%, *Anopheles lesteri *1.0%, and *Anopheles sineroides *0.8%. *Anopheles sinensis*, *pullus*, *and lesteri *were found most frequently in rice paddies followed by: ditches, flooded areas, ground pools, wheel tracks, swamps, irrigation canals, and stream margins. *Anopheles sineroides *was found most commonly in flooded areas. Supervised classification was conducted on a LANDSAT 7 ETM+ image to identify 5–6 land-use classes that were considered to be of interest. The spatial distribution of the larval mosquito collections was overlaid on the land-use image derived from the LANDSAT image, and the number of mosquitoes habitats in each class calculated. On Ganghwa Island 67% of the immature habitats containing *Anopheles sinensis *were in the Paddy land-use class, although the class only represented 17% of the land area. In Paju District 53% of the immature habitats containing *Anopheles sinensis *were in the Paddy land-use class which represented only 9 % of the area of the district. There was significant (p < 0.05) correlation between habitats containing all four species and land use on Ganghwa Island; however, only *Anopheles sinensis *was significantly correlated with land-use in Paju District. In Ganghwa Island almost 50% of the *Anopheles sinensis *adults were collected in the paddy land-use class, and there was a positive correlation between larval and adult distributions. However, in Paju District adult *Anopheles sinensis *adults were most commonly collected in the Bare land-use class and only 17% collected in the Paddy class. There was a negative correlation between larval and adult distributions.

**Conclusion:**

Immature habitats most commonly associated with *Anopheles sinensis*, *Anopheles pullus*, *Anopheles sineroides and Anopheles lesteri *were flooded rice paddies. Adult *Anopheles sinensis*, the most commonly collected anopheline, were found closely associated with rice habitats on Ganghwa Island but not in Paju District. Both QuickBird and LANDSAT satellite data were used to display spatial data in the form of geographic coverage and descriptive information in the form of relational databases associated with the mapped features. Supervised classification of LANDSAT imagery permitted good separation between Paddy, Forest, and Water land-use classes. The immature collections of *Anopheles sinensis *were significantly correlated with land-use as determined in the land-use classification in both Ganghwa Island and Paju District. These data suggest that classified remotely sensed data could potentially be used to estimate the distribution of immature and adult mosquito populations in the Republic of Korea.

## Background

Human malaria is a complex disease and its incidence is a function of the interaction between the *Anopheles *mosquito vector, the parasite, humans, and the environment. Different mosquito species have different habitat preferences e.g. rice fields, plantations, forests, forest fringes, foothills, etc., and many of these features can be identified by satellite images [1-3]. The physical environment also plays a significant role in the distribution of species in particular geographical areas [4]. In the Republic of Korea (ROK) *Plasmodium vivax *malaria reemerged in 1993 [5], with more than 1600 cases occurring in 1997 [6].

Most species of *Anopheles *in temperate East Asia are part of the Hyrcanus group of the subgenus *Anopheles*. *Anopheles anthropophagus *is the most important vector of *P. vivax *in China [7] and may possibly be the most important vector in the ROK; however, the status of vectors in the ROK is far from clear. *Anopheles sinensis *is also known to be a vector in both China and Korea, though it is considered a less competent vector than *Anopheles anthropophagus *[8]. Both *Anopheles anthropophagus *and *Anopheles sinensis *enter human habitation readily but *Anopheles sinensis *usually leaves quickly after feeding. *Anopheles sinensis *is considered a zoophilic species [7] but will readily feed on humans as well [9]. There is a possibility that *Anopheles sinensis*, which occurs widely throughout much of Asia, is composed of many species. *Anopheles sinensis *has been found in Thailand but there is inadequate evidence to ascertain its status as a malaria vector. However, a recent study indicates that *Anopheles sinensis *from Thailand is genetically similar to the Korean strain [10]. *Anopheles yatsushiroensis *and *Anopheles lesteri *are both reported in Korea, and are suspected of being vectors because they both bite humans, though neither of these species have ever been found infected with malaria. Complicating the situation further in Korea is that *Anopheles yatsushiroensis *appears to be conspecific with *Anopheles pullus*, and it is also possible that *Anopheles lesteri *and *Anopheles anthropophagus *are the same species. In this study we follow the nomenclature described in 1998 by Lee et al. [11].

Since passive instruments on satellites measure only radiation (reflected light or emitted energy) the basic products commonly extracted from these satellite observations include: vegetation index, surface temperature and cloud temperature. These parameters are widely used to determine the ecological conditions on the ground [12] and assess information on ecological variables relevant to planning of malaria control [2,3,13]. Low spatial resolution satellite data such as those from NOAA (National Oceanographic and Atmospheric Administration) and Meteosat, are available at a scale and frequency of observations that they can frequently cover large remote areas. NOAA satellite data are commonly used to monitor changing ecological conditions over multiple years because an inter-calibrated normalized difference vegetation index data set is available from 1981 to the present [14-16]. High spatial resolution satellite data (such as LANDSAT, IKONOS, and QuickBird) can be used to provide up-to-date detailed digital data that can be used as a source of spatial baseline mapping [1-3,7]. These high-resolution data can be used at various times over intervals of several years to create precise land-use maps that can serve as basis for the interpretation of both low-resolution satellite data and other geographical socio-economic data. The spatial resolution of a particular satellite sensor is important because the user must determine which data are appropriate for a particular application. QuickBird, with an extremely high resolution of 60 cm (pixel size of 60 cm by 60 cm), and LANDSAT Thematic Mapper, with a high spatial resolution of 30 m (pixel size of 30 m by 30 m), can be used to map objects in detail and characterize land use in an area [17]. Recent development in the access of remotely sensed vegetation and weather data, and their analysis along with other data sources within a GIS, have opened new possibilities in malaria stratification, monitoring, and early warning.

To better assess the risk of malaria transmission we conducted a surveillance study to identify and characterize the habitats that produce potential *Anopheles *vector mosquitoes. Immature and adult mosquito collection data were incorporated into a Geographic Information System (GIS) along with remotely sensed satellite imagery, and imagery classified to land use to determine if remote sensing data could be used to estimate mosquito habitats.

## Methods

### Study area

Study sites on Ganghwa Island and in neighboring Paju District, Republic of Korea were chosen because they exhibited documented consistent and reoccurring malaria transmission. Mosquito collections, site-mapping, and verification of land-use calculations were conducted on 3 occasions between 2 August and 28 September 2003 at the following times and places:

1. During the period from 2 August – 14 August 2003, surveys were conducted in endemic areas of Songhae-myeon and Gangwa-eup, of Ganghwa-gun, Incheon Metropolitan City, ROK. Study areas in Songhae-myeon were Dangsa-ri, Shangto1-ri, Shangto2-ri, Shindang-ri, Soljuang-ri, and Hato-ri. Study areas in Ganghwa-eup were Daesan-ri, Wolgot-ri, Namsan-ri, Yougjung-ri, and Oaknim-ri.

2. During the period from 31 August – 12 September 2003, surveys were conducted in Paju-gun, Gyeonggi-do, ROK. Study areas included Munsan-eup (Majeong2-ri), Papyeong-myeon (Jangpa1-ri and Kumpa2-ri), Jeokseong-myeong (Kawolli), Tanhyeon-myeon (Ogeum1-ri), Wollong-myeon (Neaugsan2-ri), Gyoha-eup (Yantasan-ri), Paju-eup (Hyangyang1-ri), Jori-eup (Tungwon-ri), Beopwon-eup (Tongmun-ri), Gwangtan-myeon (Changman2-ri), Geumchon1-dong, and Geumchon2-dong except Gunnae-myeon since it is a military area.

3. During the period from 21 – 28 September 2003, surveys were conducted in Paju-gun, Gyeonggi-do, ROK. Study areas included Munsan-eup (Samok-ri and Majong1-ri), Papyeong-myeon (Jangpa-ri), Wollong-myeon (Dookun-ri), Gyoha-eup (Ohdo-ri), Paju-eup (Bongseo-ri), Beopwon-eup (Gewngok-ri), and Gwangtan-myeon (Boonsoo-ri).

### Larval collections

Larval collections were made in and around each of the study areas to determine the types and abundance of habitats where anophelines occur. Collections consisted of systematically dipping with plastic dippers (0.5 liter). Geographical coordinates for all larval habitats were recorded using a Global Positioning System (GPS) GeoExplorer III with a spatial margin of error of 5 meters. Mosquito larvae were reared to adults, and identified to species [11].

### Adult mosquito collections

**Figure 2:**
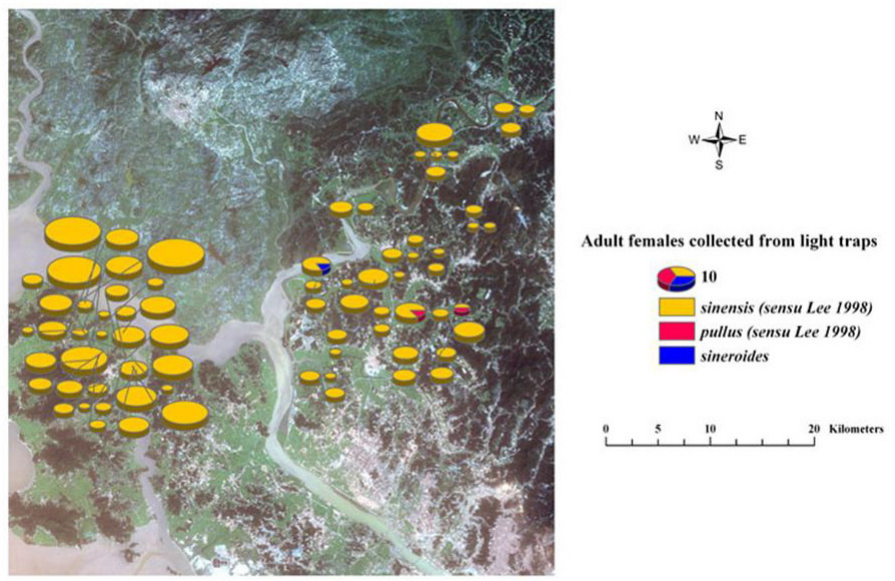
**Adult females collected in light traps**. Thematic map shows total numbers of adult anophelines collected in light traps during 32 nights during August-September 2003 overlaid on LANDSAT 7 satellite data (dated 10 September 2002, spatial resolution 30 × 30 meters) displayed in true color (band combination 3,2,1).

Three CDC-type miniature light traps were set each night outside houses, which were located in the vicinity of the larval survey. A total of 39 traps were set on Ganghwa Island during 13 nights of collections (2 August – 14 August 2003) and 36 traps were set in Paju District during 12 nights of collection (31 August – 12 September 2003) and 21 traps were also set in Paju District during 7 nights of collection (21 – 28 September 2003). Additional adult mosquito collections were conducted in cowshed using backpack aspirators; however, these data are not included in Figure 2. Mosquitoes of genera other than *Anopheles *were discarded and not considered further in this study. Adult *Anophleles *specimens were identified to species [11]. The ovaries were examined to determine parity status [18].

### Satellite data

The satellite data included a single QuickBird image (acquired 11 May 2003) with a ground resolution of 60 × 60 cm for a panchromatic image and 1 × 1 m for a multispectral image, and a LANDSAT 7 ETM+ enhanced Thematic Mapper images (acquired 10 September 2002) with a ground resolution of 30 × 30 m. These data were processed using ERDAS (2002) [19] and ENVI (2000) [20] softwares, and were used to establish the GIS for the study area. Thematic maps were generated using ArcGIS 8.3 (ESRI 2001–2004) [21] software. A RS-based GIS included spatial and temporal data in the form of geographic coverage, and descriptive information in the form of relational databases associated with the mapped features. Additional LANDSAT 7 ETM+ for coinciding with mosquito collections 2003 were not acquired due funding limitations.

### Geometric correction

A LANDSAT 7 ETM+ data set (Path 116 and Row 34) covering Ganghwa Island and Paju District in South Korea was processed and analyzed to generate land-use/land-cover information. Two subsets, one of Ganghwa Island and another of Paju District, were made from the full scene of LANDSAT 7 ETM+ data. Ground control points (GCPs), selected from the subsets and their corresponding geographic coordinates were determined by GPS survey in the field. Six and 5 GCPs were selected to geometrically correct the Ganghwa Island and Paju District subsets of 10 September 2002, respectively.

### Image-to-map registrations

Using the 6 and 5 GCPs on Ganghwa Island and Paju District, respectively, image-to-map registrations were performed to reference the Ganghwa and Paju subsets of 10 September 2002 to a UTM projection. RST (rotation, scaling and translation) warp methodology and nearest neighbor resampling were applied. Using the geo-corrected subset images of 10 September 2003 as base images, the image subsets from the other 3 LANDSAT data were referenced using image-to-image registration. RST warp methodology and nearest neighbor resampling were applied. A sub-pixel accuracy of the registration process was achieved. The Root mean square errors of the registration process were 0.31 and 0.13 for the Ganghwa Island and Paju District subsets, respectively.

### Land-use/land-cover classification

Supervised classification was performed to cluster pixels in the subset image data into land-use/land-cover classes. This was done by defining regions of interest (ROI) that represented each of the 6 or 5 desired land-use/land-cover classes in the output image corresponding to possible immature *Anopheles *mosquito habitats on Ganghwa Island and Paju District, respectively. In both study areas ROI's included: Rice paddy, Forest, urban, bare land, and water. On Ganghwa Island ROI's also included Seawater class. Utmost attention was made in selecting ROI's that are homogeneous by exporting them to n-D Visualizer function of ENVI 3.4 [20] image processing software and correcting for overlaps between classes. After the ROIs are finalized, maximum likelihood classification is performed to assign each pixel in the subset image data to the class that has the highest probability.

### Statistical analysis

Correlations between the distribution of *Anopheles *species immature habitats, adult habitats, and land-use classes, as developed from a supervised classification of LANDSAT image data, were determined by calculation of the Pearson correlation coefficients with two-tailed observed significance levels less than 0.05 [22].

## Results

Between August-September 2003 a total of 2,160 larval anopheline mosquitoes were collected from 186 larval habitats categorized into 9 types (Table 1). The collections contained 4 species of *Anopheles*: *Anopheles sinensis *representing 97.3% of the specimens, *Anopheles pullus *1.0%, *Anopheles lesteri *1.0% and *Anopheles sineroides *0.7%. *Anopheles sinensis *was found most frequently in a rice paddy and then in order of number of larvae in the following habitats: ditch, flooded area, ground pool, wheel track, swamp, irrigation canal, stream margin. *Anopheles pullus *also was found most frequently in a rice paddy followed by ditch and flooded habitats. *Anopheles sineroides *was found most commonly in flooded areas, followed by ground pool, ditch, and rice paddy habitats. *Anopheles lesteri *was found most often in a rice paddy, followed by ground pool, ditch, flooded area, and wheel track habitats.

**Table 1:** Immature habitats of anopheline mosquitoes collected during August-September 2003;

Immature Habitat	*Anopheles sinensis*^a^	*Anopheles pullus*	*Anopheles lesteri*	*Anopheles sineroides*
Ditch	374 (18)	8 (36)	2 (10)	2(14)
Flooded area	132 (6.3)	1(4)	2 (10)	7(50)
Ground pool	128 (6.1)	0 (0)	3 (13)	3(22)
Irrigation canal	81 (3.9)	0 (0)	0(0)	0(0)
Plastic container	1 (0.1)	0 (0)	0(0)	0(0)
Rice paddy	1162 (55.3)	13 (60)	14 (63)	2(14)
Stream margin	12 (0.1)	0 (0)	0(0)	0(0)
Swamp	105 (5.1)	0(0)	0(0)	0(0)
Wheel track	107 (5.1)	0(0)	1(4)	0(0)
Total collected	2,102	22	22	14

^a^The number of larvae collected (% of total collected).

**Figure 1:**
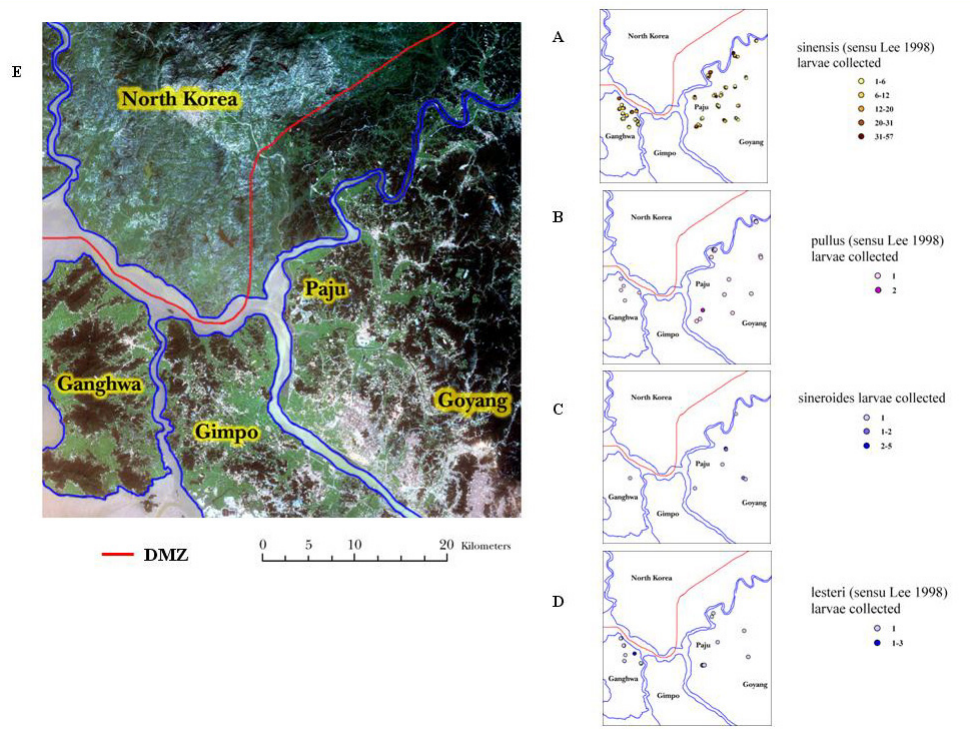
**Thematic map showing numbers of anopheline larvae collected at various habitats**; Shown are *Anopheles sinensis *in A, *Anopheles pullus *in B, *Anopheles sineroides *in C, and *Anopheles lesteri *in D, surveyed during August-September 2003, and LANDSAT 7 satellite data (dated 10 September 2002, spatial resolution 30 × 30 meters) displayed in true color (band combination 3, 2,1) in E.

The spatial distribution of larval habitats in the vicinity of the study area is presented in Figure 1. The figures represent thematic maps showing the number of larvae for each species of *Anopheles *collected at a particular site. These larval collection data are displayed on a LANDSAT satellite image displayed in true color (Band combination 3, 2, 1). In these images dark green, light green, gray, and blue-gray represent forests, rice paddies, villages/urban areas, and streams/rivers, respectively. *Anopheles sinensis *was commonly found in rice paddies near river systems and forest margins on both Ganghwa Island and Paju District. It was sometimes collected within forest areas at lower numbers. *Anopheles pullus *was widely distributed in Paju District and less so on Ganghwa Island. It was commonly found along the margins of rice paddies and never within forest areas. *Anopheles sineroides *was found in several locations in Paju District in rice paddies and along streams, and only collected in one location near a village on Ganghwa Island. *Anopheles lesteri *was found equally distributed on Ganghwa Island and Paju District in rice paddies and some forest area.

**Figure 3:**
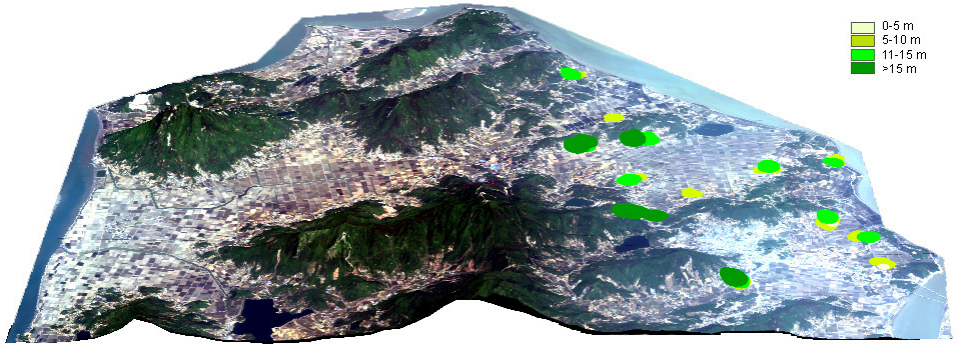
**Locations of larval habitats of North Ganghwa Island**. Collection sites are displayed on a true color of QuickBird multispectral satellite image (resolution 1 × 1 m.) overlaid on the Digital Elevation Model to depict 3-dimensions. Collection sites are defined by various shades of green which depict a range of elevations in meters above mean sea level (pale yellow = 0–5 meters, lime green 5–10 meters, florescent green 11–15 meters, and dark green >15 meters. Colored areas represent habitats that contained anopheline larvae during 2–14 August 2003.

A total of five hundred anopheline females were captured during 32 nights of light trap collections. The spatial distributions of the adult collections are shown in Figure 2. The number of *Anopheles *collected per trap night was almost twice as high in Ganghwa Island than in Paju District. The collections contained 3 species of *Anopheles*: *Anopheles sinensis *representing 98.8% of the collection, *Anopheles pullus *0.4%, *and Anopheles sineroides *0.4%. *Anopheles sinensis *was the only species found on Ganghwa Island as adults. *Anopheles sineroides *represented about 25% of the specimens at a trap located along the Im Chan River in Paju District, but was otherwise absent from light trap collections. *Anopheles pullus *was found in significant numbers at light traps located > 5 kilometers from the Im Chan River in Paju District. Figure 3 displays locations of larval habitats of North Ganghwa Island on a true color of QuickBird multispectral satellite image (resolution 1 × 1 m.) overlaid on the Digital Elevation Model (DEM) to depict 3-dimensions. Areas colored in various shades of green depict different elevations for immature habitats of anopheline larvae during 2–14 August 2003

**Figure 4:**
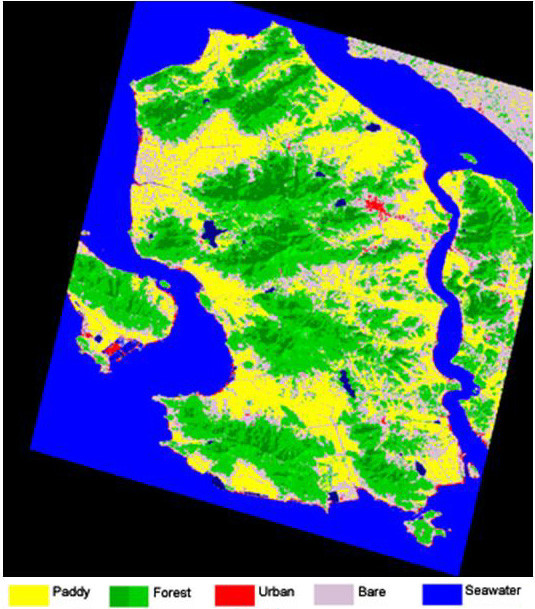
**Land-use/land-cover classification of Ganghwa Island**. Image was acquired on 10 September 2002. Rice paddy, forest, urban, bare land, seawater, and inland water land-use are depicted by yellow, green, red, gray, light blue, and dark blue, respectively.

**Figure 5:**
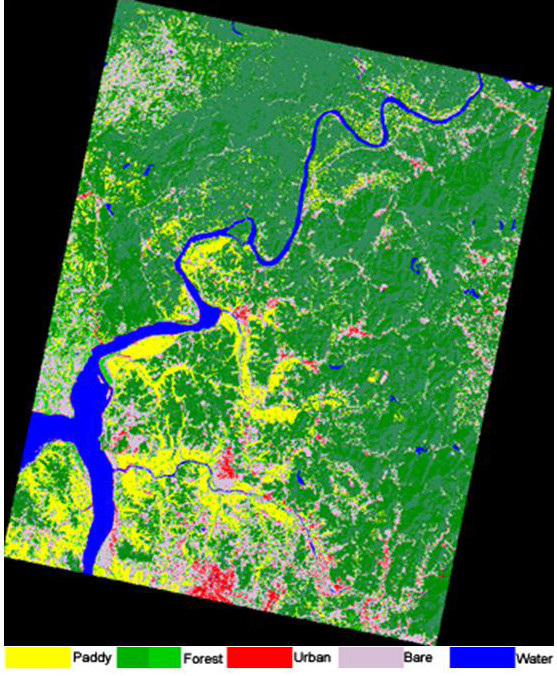
**Land-use/land-cover classification of Paju District**. Image was acquired on 10 September 2002. Rice paddy, forest, urban, bare land, and water are depicted by yellow, green, red, gray, and blue, respectively.

Land-use/land-cover classes of Ganghwa Island and Paju District are depicted in false color composites in Figures 4 and 5, respectively. These land-use classes were calculated from a satellite image data acquired on 10 September 2002, and are summarized below and in Table 2 and 3. Distribution data for *Anopheles *sp. larvae and adults in different land-use classes are shown in Tables 2 and 3, respectively. Tables 4 and 5 show confusion matrices for Ganghwa Island and Paju District, respectively, which compare land-use class derived from the satellite image data to ground truth observations presented as the number of pixels correctly classified.

**Table 2:** Distribution of *Anopheles *sp. larvae in different land-use classes on Ganghwa Island and Paju District, Republic of Korea.

Land-use/land cover	Area(ha)^a^	*Anopheles sinensis*^b^	*Anopheles pullus*	*Anophelessineroides*	*Anopheles lesteri*
**Ganghwa Island **A,B,C,D		A,B	A,C	A,B,C,D	B,C,D
Paddy	11218 (17)	44 (67)	2 (40)		5 (46)
Forest	20992 (32)	17 (24)	2 (40)	1	3 (27)
Urban	640 (1)				
Bare land	11397 (18)	9 (13)	1 (20)		3 (27)
Seawater	20637 (31)				
Inland water	399 (1)				
TOTAL	65286	70	5	1	11
					
**Paju District **A		A,B	B,C	B,C,D	D
Paddy	11577 (9)	61 (53)	8 (50)	5 (71)	5 (62)
Forest	80606 (65)	33 (29)	4 (25)	1 (14)	1 (12)
Urban	1927 (2)				
Bare land	23641 (19)	21 (18)	4 (25)	1 (15)	2 (12)
Water	5718 (5)				
TOTAL	123471	115	16	7	8

Land-use classes were created from a LANDSAT 7 ETM+ image acquired on 10 September 2002; ^a^Areas are indicated in hectares (% of total area). ^b ^The number of sites (% of sites) containing larvae of each species of mosquitoes are indicated for each land-use. Mosquitoes collected during August-September 2003. For each of the 2 study areas significant correlation coefficients at the 0.05 level (2 tailed) are indicated by the same capital letters for Land-use, Area and mosquito species.

**Table 3:** Distribution of *Anopheles *sp. adults in different land-use classes on Ganghwa Island and Paju District, Republic of Korea.

Land-use/land cover	Area(ha)^a^	*Anopheles sinensis*^b^	*Anopheles pullus*	*Anophelessineroides*	*Anopheles lesteri*
**Ganghwa Island**					
Paddy	11218 (17)	16 (47) [15]			
Forest	20992 (32)	7 (21)[7]			
Urban	640 (1)				
Bare land	11397 (18)	11 (32)[10]			
Seawater	20637 (31)				
Inland water	399 (1)				
TOTAL	65286	34 [32]			
					
**Paju District**	A	A	B	B,C	B,C
Paddy	11577 (9)	7 (17) [6]	8 (50) [1]	5 (71)	5 (62)
Forest	80606 (65)	11 (27) [12]	4 (25)	1 (14)	1 (12)
Urban	1927 (2)	1 (3) [1]			
Bare land	23641 (19)	21 (53) [14]	4 (25)	1 (15) [1]	2 (12)
Water	5718 (5)				
TOTAL	123471	40	16	7	8

Land-use classes were created from a LANDSAT 7 ETM+ image acquired on 10 September 2002; ^a^Areas are indicated in hectares (% of total area). ^b ^The number of sites (% of sites) [number of parous females] where adults of each species of mosquitoes were collected are indicated for each land-use. Mosquitoes collected during August-September 2003. For each of the 2 study areas significant correlation coefficients at the 0.05 level (2 tailed) are indicated by the same capital letters for Land-use, Area and mosquito species.

**Table 4:** Confusion matrix of the LANDSAT (10 September 2003) classified image of Ganghwa island, Republic of Korea.

CLASS	GROUND TRUTH (PIXELS)						
	
	Inland water	Bare land	Urban	Paddy	Forest	Seawater	Total
Inland water	1023	0	0	0	0	0	1023
Bare land	106	441	141	49	13	28	778
Urban	5	41	341	0	0	38	425
Paddy	2	11	0	975	2	0	990
Forest	11	1	0	23	1078	0	1113
Seawater	20	0	0	0	0	992	1012
Total	1167	494	482	1047	1093	1058	5341

Agreement between classified image classes and ground truth observation is based upon an assessment of the classified image. Overall Accuracy = 90.8%; Kappa Coefficient = 0.89

**Table 5:** Confusion matrix of the LANDSAT (10 September 2003) classified image of Paju District, Republic of Korea.

CLASS	GROUND TRUTH (PIXELS)					
	
	Water	Bare land	Urban	Paddy	Forest	Total
Water	3820	1	9	0	0	3830
Bare land	42	958	412	13	13	1432
Urban	26	50	589	0	0	665
Paddy	0	4	0	3086	3086	3095
Forest	0	36	4	200	200	4264
Total	3888	1049	1014	3299	3299	13286

Agreement between classified image classes and ground truth observation is based upon an assessment of the classified image. Overall Accuracy = 93.9%; Kappa Coefficient = 0.9186

### Ganghwa Island (Figures 3,4; Tables 2, 3,4)

In the more than 65,000 hectares on Ganghwa Island we identified 6 land-use classes (Figure 4). The overall accuracy of the classification on Ganghwa Island was 90.8% and the Kappa coefficient = 0.8880 based upon ground truth observations (Table 4). Forest and Seawater classes were predominant, each exceeding 30% of the total area of the island. Good separation between Paddy, Forest, and Water land-use classes was achieved. However, separation between Urban and Bare land was poor, since these classes overlap significantly in their spectral signatures. In the classified image paddy fields with standing rice crop appear "yellow" on the image and can be easily identified from forests, which appear green, and from water classes (blue). Urban areas of Ganghwa Island can be identified, but are confused to some extent with bare land. A few paddy fields without standing water are classified as bare lands. Forest areas are identified reasonably well in all datasets.

The spatial distribution of the larval and adult mosquito collections was overlaid on the land-use image derived from the LANDSAT image, and the number of mosquitoes habitats identified in each class calculated. On Ganghwa Island 67% of the immature habitats containing *Anopheles sinensis *were in the Paddy land-use class, although the class only represented 17% of the total land area (Table 2). There was significant (p < 0.05) correlation between habitats containing all four species and land use on Ganghwa Island.

Almost 50% of the *Anopheles sinensis *adults were collected in the Paddy land-use class, followed by Bare land and Forest classes, and parous individuals were found in each of the land-use classes (Table 3). There was a correlation between adult *Anopheles sinensis *and land use but this was not significant (p = 0.78). The positive correlation between larval and adult distributions was not significant.

### Paju District (Figure 5; Tables 2,3, 5)

In the more than 123,000 hectares in Paju District we identified 5 land-use classes. The overall accuracy of the classification in Paju District was 93.9% and the Kappa coefficient = 0.9186 based upon ground truth observations (Table 5). The Forest land-use class predominated, exceeding 65% of the total area in Paju District. Similar to the observations made on Ganghwa Island, paddy fields with standing rice crop are easily identified as "yellow" color in the false color composite of 10 September 2002 (Figure 5).

Table 2 summarizes the land-use/land-cover classification results of Paju District. In Paju District 53% of the immature habitats containing *Anopheles sinensis *were in the Paddy land-use class which represented only 9% of the area of the district. The spatial distribution of the larval and adult mosquito collections was overlaid on the land-use image derived from the LANDSAT image, and the number of mosquitoes habitats identified in each class calculated. There was significant (p < 0.05) correlation between land-use classes and habitats containing *Anopheles sinensis*. The positive correlation between the other 3 species found in Paju District and land-use was not significantly correlated. There was a strong and significant correlation between the distributions of each of the species.

Adult *Anopheles sinensis *adults were most commonly collected in the Bare land class, followed by the Forest class and only 17% collected in the Paddy class (Table 3). Parous individuals were found in Paddy, Forest, urban and Bare land classes, and there was a strong and significant correlation between adult *Anopheles sinensis *and land area (p < 0.05). There was a strong negative correlation between the distribution of larvae and adult *Anopheles sinensis*, although the correlation was not significant (p = 0.109).

*Anopheles pullus*, *Anopheles sineroides *and *Anopheles lesteri *adults were found primarily in the Paddy class, and there distributions were significantly correlated. For each of these species there was a strong significant (p < 0.05) correlation between larval and adult collection habitats.

## Discussion and conclusions

This study explored the spatial distribution of the immature and adult stages of *Anopheles *mosquitoes that are considered to be important vectors of *Plasmodium vivax *in the Republic of Korea [23,24]. *Anopheles sinensis *was the most commonly collected anopheline species. In 1997 Strickman et al. [9] also found *Anopheles sinensis *was the most commonly collected adult mosquito followed by *Anopheles lesteri *and *Anopheles pullus *(reported as *Anopheles yatsushiroensis*).

Mosquito control is most efficient when it targets immature stages; however, successful control strategies are dependent upon the ability to identify habitats for these stages [25]. Rice paddies were the most commonly associated habitats with larval *Anopheles sinensis*, *Anopheles pullus*, *Anopheles sineroides and Anopheles lesteri *in both Ganghwa Island and Paju District. In Paju District there was a negative correlation between the habitats for larvae and those for adults, suggesting that adults are leaving rice paddy areas to seek human blood. This type of adult mosquito behavior may have an impact on decisions regarding how, when and where to conduct larval control since previous studies have suggested that larval control may be effective in controlling malaria transmission in Korea [26].

Immature stages of *Anopheles sinensis *mosquitoes are found in a variety of aquatic habitats and under a variety of environmental conditions [4]; however, larval surveys are often very expensive, time-consuming, and cumbersome. As an alternative to a traditional ground-based survey approach to vector surveillance, we evaluated the potential for application of remote sensing (RS) data in a Geographic Information System (GIS) to identify land-use classes that are correlated to anopheline habitats. Such information could be used to assess the risk of human – vector contact, and resulting malaria transmission risk. Remote sensing has been used to estimate rice yields [27], and identify and monitor rice-field development [28]. The accuracy of GIS and remote sensing technologies in estimating the identity of larval and adult habitats that produce key *Anopheles *mosquito vector species was evaluated by comparing data collected by ground observations to classified images. Both QuickBird and LANDSAT satellite data were used to successfully display spatial data in the form of geographic coverage and descriptive information in the form of relational databases associated with the mapped features. Supervised classification of LANDSAT imagery permitted good separation between paddy, forest, and water land-use classes. The immature collections of *Anopheles sinensis *were significantly correlated with land-use as determined in the land-use classification in both Ganghwa Island and Paju District. There was also a correlation between the spatial distribution of adult *Anopheles sinenesis *and land-use. These data suggest that classified remotely sensed data could potentially be used to estimate the distribution of immature and adult mosquito populations in the Republic of Korea.

To better asses malaria risk factors further studies are required to address topics that include: 1) testing mosquito specimens from man-biting collections for the presence of malaria parasite using molecular techniques to determine vectorial capacity and entomological inoculation rates, 2) performing proximity analysis of each patient from immature mosquito habitats to quantify risk of living in areas with *Anopheles *immature habitats, and 3) performing detailed GIS analysis with the input of biophysical ground truthing and meteorological parameters from climate satellites to define the temporal dynamics of malaria transmission. It is anticipated that in the future the GIS databases will be used to quantify spatial and temporal relationships between larval habitats and characterization of adult mosquito density in the associated villages. Final products of the GIS will include maps depicting the spatial distribution of larval mosquito habitats for various species and a map depicting potential adult mosquito population in villages proximal to the immature habitats.

## Authors' contributions

All authors have participated fully in the conduct of this study, the acquisition of data, the analysis and/or the preparation of the manuscript in a substantial manner. RS and KJL performed the statistical analysis and drafted the manuscript; WJL and DMU provided critical review and input. RS, WJL, DMU, and KJL participated in the design of the study, participated in interpretation. Field data acquisition efforts were conducted by RS and WJL. All authors read and approved this manuscript.
